# From Cell to Gene: Deciphering the Mechanism of Heart Failure With Single‐Cell Sequencing

**DOI:** 10.1002/advs.202308900

**Published:** 2024-08-19

**Authors:** Dan Zhang, Qiang Wen, Rui Zhang, Kun Kou, Miao Lin, Shiyu Zhang, Jun Yang, Hangchuan Shi, Yan Yang, Xiaoqiu Tan, Shigang Yin, Xianhong Ou

**Affiliations:** ^1^ Key Laboratory of Medical Electrophysiology of Ministry of Education Institute of Cardiovascular Medicine Department of Cardiology of the Affiliated Hospital Southwest Medical University Luzhou Sichuan 646000 China; ^2^ Department of Rehabilitation Medicine Southwest Medical University Luzhou Sichuan 646000 China; ^3^ Department of Cardiology Union Hospital Tongji Medical College Huazhong University of Science and Technology 1277 Jiefang Rd Wuhan Hubei 430022 China; ^4^ Department of Clinical & Translational Research University of Rochester Medical Center 265 Crittenden Blvd Rochester NY 14642 USA; ^5^ Department of Pathology and Laboratory Medicine University of Rochester Medical Center 601 Elmwood Ave Rochester NY 14642 USA; ^6^ Department of Physiology School of Basic Medical Sciences Southwest Medical University Luzhou Sichuan 646000 China; ^7^ Luzhou Key Laboratory of Nervous system disease and Brain Function Southwest Medical University Luzhou Sichuan 646000 China; ^8^ State Key Laboratory for Chemistry and Molecular Engineering of Medicinal Resources Guangxi Normal University Guilin Guangxi 541004 China

**Keywords:** application, challenging, heart failure, mechanism, single‐cell sequencing

## Abstract

Heart failure (HF) is a prevalent cardiovascular disease with significant morbidity and mortality rates worldwide. Due to the intricate structure of the heart, diverse cell types, and the complex pathogenesis of HF, further in‐depth investigation into the underlying mechanisms  is required. The elucidation of the heterogeneity of cardiomyocytes and the intercellular communication network is particularly important. Traditional high‐throughput sequencing methods provide an average measure of gene expression, failing to capture the “heterogeneity” between cells and impacting the accuracy of gene function knowledge. In contrast, single‐cell sequencing techniques allow for the amplification of the entire genome or transcriptome at the individual cell level, facilitating the examination of gene structure and expression with unparalleled precision. This approach offers valuable insights into disease mechanisms, enabling the identification of changes in cellular components and gene expressions during hypertrophy associated with HF. Moreover, it reveals distinct cell populations and their unique roles in the HF microenvironment, providing a comprehensive understanding of the cellular landscape that underpins HF pathogenesis. This review focuses on the insights provided by single‐cell sequencing techniques into the mechanisms underlying HF and discusses the challenges encountered in current cardiovascular research.

## Graphical abstract

This review summarizes the current applications of single‐cell sequencing (SCS) in elucidating the mechanisms of heart failure (HF), including energy metabolism, inflammation, ischemia, extracellular matrix remodeling, and small molecular mediators. It emphasizes cardiomyocyte heterogeneity and intercellular communication, providing insights into gene expression alterations and distinct cell populations within the HF microenvironment, and highlights the importance of SCS for future HF research and treatment strategies.

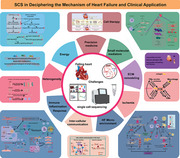

## 1 Introduction

Heart failure (HF) is the end stage of many cardiac diseases and a severe process of cardiac dysfunction. The irreversibility of cardiac cell damage makes treating cardiac conditions, especially HF, extremely challenging. A variety of heterogeneous cells finely integrate the heart. Understanding the developmental processes, cell types, and gene expression patterns of various cell types at the cell level is more conducive to precisely treating HF. Single‐cell sequencing (SCS) has undergone significant development in recent years. The first single‐cell RNA sequencing (scRNA‐seq) method was developed in 2009,^[^ ^1^ ^]^ but until 2015, scRNA‐seq techniques found widespread application. Since then, several methods for SCS have been developed, including DNA and RNA sequencing. These advances have opened up new opportunities for researchers to explore previously unknown cell types, hidden patterns of gene expression, and the effects of genetic mutations at the single‐cell level, making it possible to deeply analyze highly heterogeneous heart tissue with HF. In addition, SCS enables cell trajectory analysis, which is essential for elucidating cell state transitions during cardiac development, progenitor cell differentiation, and predicting intercellular communication for ligand‐receptor binding based on gene expression, providing new insights into disease occurrence, outcome, and treatment.

## 2 The Basic Process of SCS Technology

**Figure 1:**
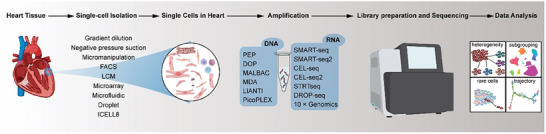
The basic flow of single‐cell sequencing. Single‐cell isolation is the process of obtaining single cells from heart tissue using multiple methods, including the gradient dilution method, negative pressure suction method, micromanipulation method, fluorescence‐activated cell sorting (FACS), laser capture microdissection (LCM), microarray technology, microfluidic techniques, and droplet technology, and the ICELL8 cx Single‐Cell System. Single‐cell amplification refers to increasing the amount of genetic material, either DNA or RNA, from a single cell to enable further analysis. Single‐cell DNA amplification replicates the genomic DNA of a single cell, while single‐cell RNA amplification replicates the RNA transcripts present in a single cell. Library construction for scRNA‐seq involves converting RNA into complementary DNA (cDNA), adding adapters, and amplifying the cDNA. The sequencing step then determines the order of nucleotides in the cDNA. Data analysis for sscRNA‐seq involves processing and interpreting the sequencing data to identify and characterize gene expression patterns at the individual cell level. This analysis includes steps such as quality control, normalization, dimensionality reduction, clustering, differential gene expression analysis, and functional annotation to gain insights into the cell populations and their biological functions.

The basic flow of SCS is shown in **Figure** **1**. The quality of cells is a prerequisite for the success or failure of SCS. So, single‐cell isolation and sample preparation are important for the whole flow. Various single‐cell isolation and extraction methods mainly including the gradient dilution method,^[^ ^2^, ^3^ ^]^ negative pressure suction method,^[^ ^4^ ^]^ micromanipulation method,^[^ ^4^, ^5^ ^]^ fluorescence‐activated cell sorting (FACS),^[^ ^6^ ^]^ laser capture microdissection (LCM),^[^ ^7^, ^8^ ^]^ microarray technology,^[^ ^9^ ^]^ microfluidic techniques,^[^ ^6^, ^10^, ^11^ ^]^ and droplet technology,^[^ ^7^ ^]^ and the ICELL8 cx Single‐Cell System,^[^ ^12^ ^]^ have been developed according to the type, state, and analysis purpose of samples. A detailed comparison of these approaches is presented in **Table** **1**. However, the dense structural organization of the heart and the presence of collagen make cardiomyocytes (CMs) particularly sensitive to hypoxia, posing significant challenges to their isolation. To address this issue in heart scRNA‐seq research, See et al. modified a well‐established microfluidic platform for single nuclear RNA‐seq (snRNA‐seq). Although cytoplasmic RNA was lost, snRNA‐seq successfully retained the nascent initial transcript types and yielded abundant non‐coding RNAs.^[^ ^13^ ^]^ The stability of nuclei compared to their cellular counterparts, while preserving the heterogeneity of CMs, has made snRNA‐seq a widely used method for SCS of the heart.^[^ ^14^ ^]^

**Table 1:** Single‐cell sample preparation methods.

Method	Main Process	Advantage	Disadvantage
The gradient dilution method	Achieve a cell suspension with a targeted cell concentration of one cell per volume unit.	Low‐cost, simple separation without specialized instruments.	High probability of blank space, multicellularity and separation errors, low efficiency, limited separation, not suitable for rare cells.
Negative pressure suction method	The oral pipette technique allows for selecting cells with optimal morphology via a microscope.	Negligible cell damage	Operator skill dependency, inability to discriminate cell types under a microscope, low flow rate.
Micromanipulation method	Manual or automatic selection, precision tools like a microscope are used under a microscope to isolate single cells by the operator.	High precision can target specific cells, mitigate	Skill‐intensive, low cell efficiency, operator skill dependency
Fluorescence‐activated cell sorting (FACS)	high pressure to sort fluorescently labeled single‐cell suspensions into charged droplets deflected and collected based on their charge, achieving cell separation.	High speed, precision, and accuracy in sorting large and complex cell samples	Affect cell viability, unsuitable for sorting small or difficult‐to‐sort cell populations, expensive equipment, complex operation.
Laser capture microdissection (LCM)	The laser cuts out desired cells and captures them on a special membrane	Fast, simple, precision, and specificity, preserve the isolated samples' structural integrity and avoid damaging the surrounding tissues	Disrupted integrity, damaged nucleic acids, impact on amplification and sequencing, require special equipment, high cost.
Microarray technology	it separates single cells from cell suspensions using an array of microwells, made with a pore size slightly larger than the cell diameter and selectively manipulated by gravity, fluid, or electric fields.	High thought and sensitivity, cost‐effective screening and analysis on a large scale.	High requirements for RNA samples, limited to the detection of rare genes and novel genes, high chip design and manufacturing costs.
Microfluidic techniques	In reaction chambers and droplet technologies, achieve single‐cell separation and sequencing with precise fluid flow control in microchannels through gravity, hydrodynamics, and electric fields.	High throughput, sensitivity, selectivity, and precision, highly reproducible, automation, and integration	complexity of equipment and operation, Demands a high level of proficiency from the operator
Droplet technology	aqueous solution and oil phase flow from different microchannels and fuse to form a “water‐in‐oil” droplet.	High speed, thought, and accuracy. Reduce the possibility of surface contamination	Complex equipment and reagents, Operator proficiency, potential errors from multiple steps, heightened contamination risk, Sample volume limitations
ICELL8 systems	Based on microfluidic chip technology, accurately dispenses 50nL into 5184 wells on a 4 cm^2 ICELL8 chip using the MultiSample NanoDispenser。	High efficiency, suitable for large‐scale separation, reducing sample‐handling artifacts, clean sequencing background, avoiding technical errors	Limited scalability, the cell capture efficiency is only 30%, expensive equipment, complex operation

Subsequently, SCS amplification is performed. The DNA content of single cells cannot meet the library building and sequencing, so there is a need to efficiently obtain unbiased linear amplification products with complete genome coverage, mainly divided into genome amplification and transcriptome amplification. Single‐cell transcriptome amplification is usually preceded by reverse mRNA into cDNA before amplification. There are several main methods for whole‐genome amplification,^[^ ^15^ ^]^ including primer extension preamplification (PEP),^[^ ^16^, ^17^, ^18^, ^19^ ^]^ degenerate oligonucleotide‐primed polymerase chain reaction (DOP‐PCR),^[^ ^19^, ^20^, ^21^, ^22^ ^]^ and ligation‐mediated PCR amplification (LM‐PCR).^[^ ^23^ ^]^ PicoPLEX and multiple annealing and looping‐based amplification cycles (MALBAC) techniques combine PCR and isothermal replacement methods. Multiple displacement amplification (MDA) techniques use a set of random primers with the same temperature under the action of Phi29.^[^ ^22^ ^]^ The linear amplification via transposon insertion (LIANTI) technique^[^ ^24^ ^]^ uses transposons to chop DNA randomly so DNA samples can be linearly amplified. Before conventional PCR, MALBAC and LIANTI techniques effectively suppressed amplification preferences and improved genome‐wide coverage at amplification by designing starting isothermal amplicons to form loops and transposons to be widely distributed in the genome. The advantages and disadvantages of these methods were discussed in Supporting Information 1.

To achieve single‐cell mRNA sequencing without significant PCR bias, increasing mRNA increments by several million‐fold may be necessary, owing to the considerably lower mRNA content in cells. Instead of libraries containing substantial amounts of ribosomal RNA (rRNA) sequences, obtaining libraries consisting of mRNA is feasible. It should be noted that more than 95% of the total RNA in cells is constituted by rRNA, with mRNA accounting for only 2%–3%. Consequently, if a non‐selective reverse transcription, amplification, library construction, and sequencing approach is adopted, most resulting sequences will not offer pertinent biological information about mRNA. As a result, PCR or multiplexed linear amplification techniques are generally employed for their amplification. SMART‐seq,^[^ ^25^ ^]^ SMRT‐seq2,^[^ ^26^ ^]^ CEL‐seq,^[^ ^27^ ^]^ CEL‐seq2,^[^ ^28^ ^]^ and STRTseq,^[^ ^29^ ^]^ which are based on full‐length RNA sequencing and high‐throughput microfluidics‐based methods, such as DROP‐seq^[^ ^30^ ^]^ and 10 × Genomics to achieve intracellular mRNA amplification. In Supporting Information 1, we described the process for representative applications.

After acquiring the SCS data, the subsequent procedure involves comprehensive analysis and data exploration of the extensive sequence information tailored to achieve specific research goals. This strategic approach allows the extraction of novel genetic information and disease‐associated molecular characteristics, thereby serving as a cornerstone of SCS research and an essential component within the research continuum. This article overviews the procedure for analyzing scRNA‐seq data, which included 1) quality Control and matrix generation, 2) standardization, 3) feature Selection, 4) dimensionality reduction, data visualization, and clustering analysis, 5) cell trajectory analysis, 6) gene differential expression, regulatory networks, and cell interactions. However, due to layout limitations, we discussed them in Supporting Information 1.

## 3 Application of SCS in Studying the Mechanisms of HF

**Figure 2:**
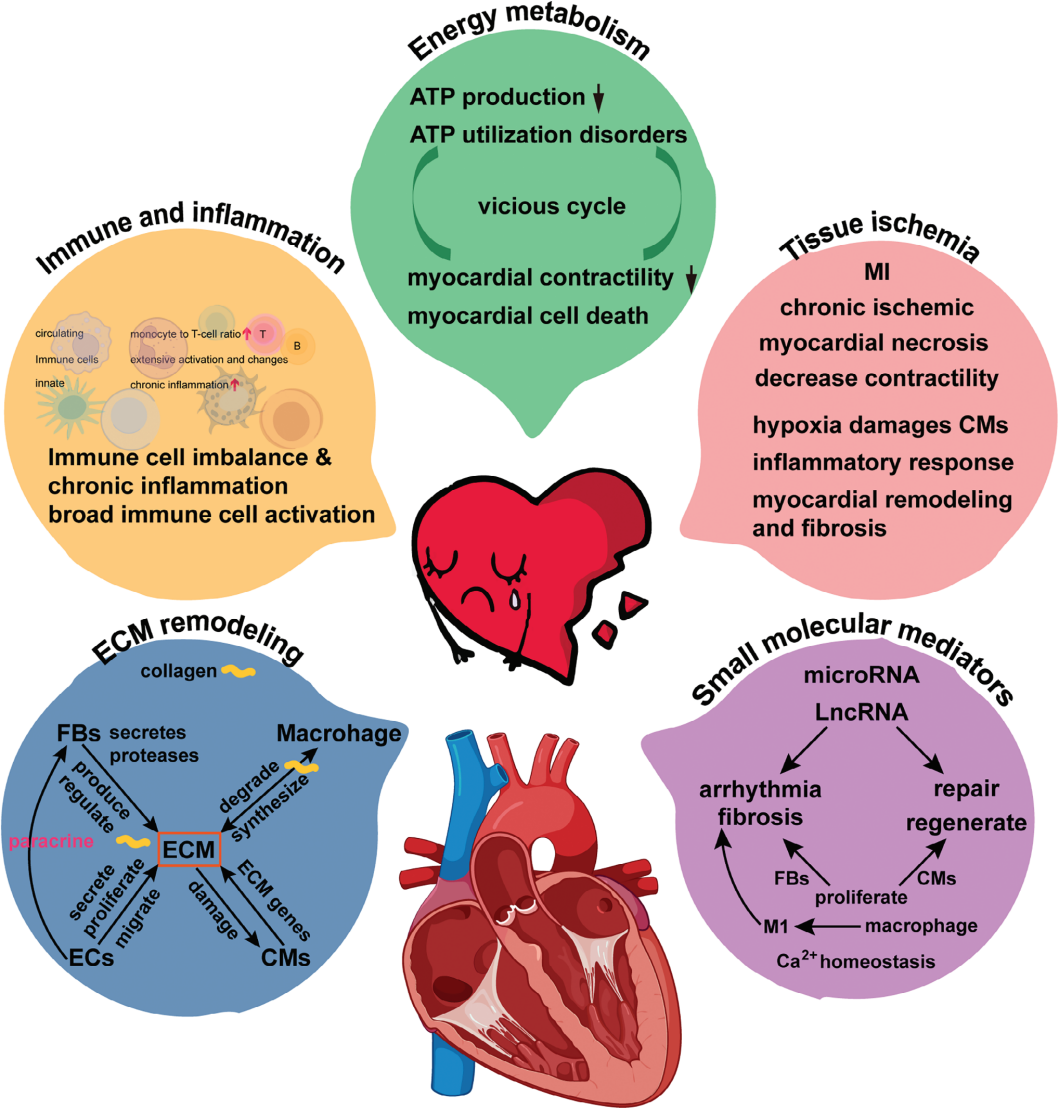
Application of SCS in studying the mechanisms of HF. With extensive use of SCS in cardiovascular, the understanding of the mechanisms of HF has deepened, mainly manifested in the following aspects: 1. energy metabolism, 2. immune and inflammation, 3. tissue ischemia, 4. ECM remodling, 5. small molecular mediators. Studying the structure and function of the heart at the single‐cell level provides reliable ideas for the study of the molecular mechanism of HF and the further treatment of heart diseases. However, we must also meet more challenges and problems with the mechanism of HF based on single‐cell studies.

HF is a complicated syndrome characterized by various functional abnormalities. During the development of HF, activation of the neurohormonal system, cardiac hypertrophy (CH), impaired energy metabolism, dysfunctional calcium handling, oxidative stress, immune cell imbalance and inflammation, and severe ischemia all cause significant changes in myocardial cell composition and status. Although extensive, conventional bulk RNA‐seq has yielded important insights into disease mechanisms of HF, average gene expression levels that are missing cell‐specific information could overlook the role of individual cell types and compromise more detailed cellular‐level mechanistic inquiry.^[^ ^31^, ^32^, ^33^ ^]^ It is essential to identify the dominant mechanism behind HF development at the cellular and molecular levels, determine the specific types of cells and genes that play a role in these alterations, and study their interactions to find critical intersections in the case of multiple mechanisms. By comprehending the localized differences within the heart, we can establish the molecular foundation and identify targets for investigating the precise mechanisms and treatments for HF. The scRNA‐seq has revolutionized our understanding of cell composition and associated gene expression by accurately scaling the average gene expression level from bulk‐RNA‐seq to the individual cell level. This technique allows us to discern cell‐to‐cell heterogeneity, even within the same cell type. Consequently, it provides a valuable technical framework for exploring the key cell populations involved in structural alterations within the heart and the underlying mechanisms of HF. Presently, an increasing number of studies are utilizing scRNA‐seq to investigate the mechanisms of HF (**Figure** **2**).

### 3.1 Impaired Myocardial Energy Metabolism in HF

**Figure 3:**
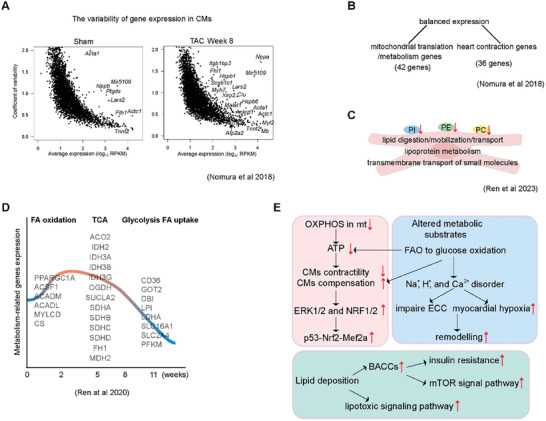
Impaired myocardial energy metabolism mediates the onset and progression of HF in several ways. A) The gene expression variability in CMs after HF. B) Cardiac morphological hypertrophy is associated with enhanced mitochondrial biosynthesis. C) The impaired intracellular metabolism of CMs in HF promotes the entry of lipid molecules into lipotoxic pathways/signaling, which further induces possible cardiac dysfunction. D) The expression of energy metabolism‐related genes mainly includes FA oxidation, TCA, and glycolysis FA uptake, which initially increases and then decreases with TAC over time. E) Mitochondrial damage leads to ATP reduction, and conversely, ATP reduction will also cause mitochondrial damage, resulting in myocardial cell death, cardiac fibrosis, and HF (Figure 3E). CMs, cardiomyocytes; TAC, transverse aorta constriction; PE, phosphatidylethanolamines; PI, phosphatidylinositol; PC, phosphatidylcholines. FA, fatty acids; FAO, fatty acids oxidation; TCA, tricarboxylic acid; OXPHOS, oxidative phosphorylation; mt, mitochondrion; ATP, adenosine 5′‐triphosphate; ECC, excitation‐contraction coupling; BACCs, branch‐chain amino acids.

Altered energy metabolism is an essential feature of HF. These mechanisms include insufficient metabolic capacity, redox imbalance, protein modification, increased ROS (reactive oxygen species) production, impaired mitochondrial Ca^2+^ homeostasis, and inflammation.^[^ ^34^, ^35^, ^36^, ^37^, ^38^ ^]^ When pathological hypertrophy and HF occur, the dynamic balance of fatty acids oxidation (FAO), aerobic glucose oxidation, and glycolysis is disrupted. With the FAO gene's down‐regulation and the glucose oxidation gene's subsequent up‐regulation, energy substrate preference is shifted from fatty acids to glucose, and mitochondrial oxidative phosphorylation (OXPHOS) is gradually decreased.^[^ ^39^ ^]^ Glycolysis is a possible way to meet the increased energy demand in the early stage of CH.^[^ ^40^ ^]^ However, glucose increases aspartate biosynthesis and provides nitrogen for nucleotide synthesis during cardiomyocyte hypertrophy, exacerbating CH.^[^ ^41^ ^]^ Thus, the FAO‐preserved metabolic approach that restores the heart to use fatty acids metabolism is more helpful in treating CH and HF.^[^ ^42^ ^]^ In a single‐cell remodeling study of the adult heart during HF and recovery, the authors described how energy metabolism affects the course of HF. Metabolic processes were significantly enriched in left ventircl (LV) CMs, and the high expression of metabolism‐related genes was consistent with the high energy demand of LV. The dysfunction of substrate metabolism leads to the disorder of myocardial metabolic pathways. The change in energy metabolism directly leads to reduced mitochondrial adenosine 5′‐triphosphate (ATP) capacity by ≈30% compared with normal myocardium, mainly due to mitochondrial damage.^[^ ^43^, ^44^ ^]^ The decrease in ATP synthesis is associated with reduced cardiac function, reduced myocardial contractility, and energy deficit, leading to processes such as glucose oxidative uncoupling and gluconeogenesis secondary to myocardial cells, resulting in disruption of intracellular Na^+^, H^+^, and Ca^2+^ ion homeostasis, which may cause a complex series of molecular mechanisms such as cellular acidosis, impaired dissociation of the myosin‐actin complex, and impaired excitation‐contraction coupling generation causing myocardial remodeling, leading to and exacerbating myocardial hypoxia. Increased free radicals in CMs lead to abnormal mitochondrial autophagy and Ca^2+^ homeostasis, further reducing ATP production and entering a vicious cycle. During compensatory CH, the initial increase in cardiac metabolism toward glycolysis can maintain normal cardiac systolic function in the short term; however, in a long time, the adaptive shift in cardiac metabolism toward increased glycolysis is insufficient to meet the energy demands of the diseased heart. In addition, increased myocardial ketone body oxidation in HF is often accompanied by decreased fatty acid oxidation. Although branch‐chain amino acids (BCAAs) have little oxidative energy supply, their impaired oxidation induces insulin resistance, and the accumulation of BCAAs activates mTOR signaling, accelerating the development of myocardial hypertrophy.^[^ ^44^, ^45^ ^]^ Furthermore, chemogenetic studies of cardiac oxidative stress suggest that increased cardiac redox stress is a central feature of HF animals.^[^ ^46^ ^]^ Similarly, the study by Nomura et al. revealed transcriptional dynamics in the remodeling trajectory of mouse CMs, identifying gene modules that distinguish CH from HF. **Figure** **3**A showed the gene expression variability in CMs after operating transverse aortic constriction (TAC) 8 weeks (HF model) compared with Sham. The balance between mitochondrial translation/metabolism genes and heart contraction genes is a native characteristic of cardiac muscle cells, while cardiac morphological hypertrophy is associated with enhanced mitochondrial biosynthesis (Figure 3B). After pressure overload, nuclear and mitochondrial translation in cardiomyocyte hypertrophy are activated synchronously to increase energy demand but cause excessive oxidative stress and accumulate DNA damage, which further mediates oxidative stress and promotes the development of HF. The activated mitochondrial translation/metabolism gene expression in CMs is associated with cell size and ERK1/2 and NRF1/2 transcriptional networks in the early stage of CH. In the late stage of hypertrophy, p53 signaling was specifically activated. Further studies showed that failure of CMs induced by p53 activation is characteristic of MEF2‐mediated cytoskeletal reorganization and NRF2‐mediated antioxidant stress response and that the p53‐NRF2‐MEF2A axis is a key factor in the induction of cardiomyocyte failure. The scRNA‐seq of CMs also confirmed these findings from HF patients.^[^ ^47^ ^]^

Lipid deposition in CMs in HF suggests a causal relationship between cardiac lipid overload and systolic/diastolic dysfunction.^[^ ^48^ ^]^ Toxic lipids or by‐products have a broad impact on the biological processes of CM and play an essential role in the progression of HF.^[^ ^49^ ^]^ Lipotoxicity, an imbalance in lipid transport and lipid metabolism, leads to CM death, cardiac injury, remodeling, and ultimately cardiac dysfunction.^[^ ^50^ ^]^ It has been shown that abnormal lipid metabolism is significantly associated with disease severity in HF with preserved ejection fraction and is a critical pathophysiological driver.^[^ ^51^, ^52^ ^]^ Panico et al. utilized scRNA‐seq to explore the impact of metabolic distress on myocardial inflammation in HF with preserved ejection fraction (HFpEF). In their mouse model driven by hyperlipidemia, heart macrophages exhibited a proinflammatory profile influenced by lipid overload and ER stress pathways. A regulatory axis between macrophages and CMs was identified, affecting pathways associated with hypertrophy, fibrosis, and autophagy.^[^ ^53^ ^]^ A single‐cell‐based lipid metabolomics study identified specific metabolic signatures of CMs in HF, revealing that these signature molecules are closely associated with lipoprotein metabolism, transmembrane transport, and signaling.^[^ ^54^ ^]^

The impaired intracellular metabolism of CMs in HF promotes the entry of lipid molecules into lipotoxic pathways/signaling, which further induces possible cardiac dysfunction (Figure 3C).^[^ ^55^ ^]^ Based on cellular interaction networks, contractility and metabolism of CMs were found to be essential features affecting the deterioration of cardiac function.^[^ ^56^ ^]^ The expression of energy metabolism‐related genes mainly includes three categories: FAO, tricarboxylic acid (TCA), and glycolysis fatty acids (FA) uptake, which initially increase and then decrease with TAC over time, as shown in Figure 3D.^[^ ^40^ ^]^ Energy metabolism disorders affect cardiac remodeling in multiple aspects, resulting in decreased cardiac function and HF. ATP production is reduced, and myocardial energy is insufficient, directly affecting the heart's contractility. The heart increases glycolysis and other pathways to compensate for the energy supply. Glycolysis and tricarboxylic acid cycle pathways produce excessive metabolites, which cause cell acidosis and further damaging CMs. The imbalance of ion homeostasis causes cardiac electrical remodeling, and the excitation‐contraction coupling disorder leads to systolic HF. After compensatory hypertrophy of the myocardium, a series of hypertrophic mechanisms lead to decompensation, and HF occurs. In this process, the increased oxygen free radicals cause oxidative damage to cell membranes, proteins, and nucleic acids, causing oxidative stress, which will lead to the activation of immune cells such as mast cells, produce a variety of inflammatory mediators, trigger inflammatory responses, and further damage CMs. Energy metabolism disorders are mainly caused by mitochondrial oxidation dysfunction. Mitochondrial damage leads to ATP reduction, and conversely, ATP reduction will also cause mitochondrial damage, resulting in myocardial cell death, cardiac fibrosis, and HF (Figure 3E).

### 3.2 Response to Immune and Inflammation Promoting the Progression of HF

**Figure 4:**
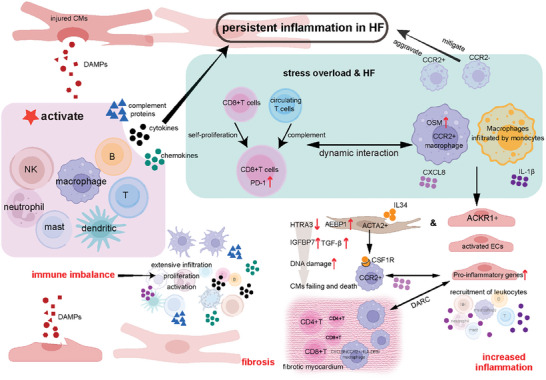
Immune cell imbalance and inflammation promote the progression of HF. The involvement of both the innate and adaptive immune systems in HF leads to localized immune responses in the heart, contributing to ventricular remodeling and dysfunction. Various types of immune response cells play a significant role in the development of HF. These cells are activated, transitioned, and regulate CMs, ECs, and FBs through complex immune and inflammatory processes during the progression of the disease. ACKR1, atypical chemokine receptor 1; ACTA2, actin alpha 2; AEBP1, AE Binding Protein 1; B, B cells; CMs, cardiomyocytes; CCR2, CC chemokine receptor 2; CSF1R, colony‐stimulating factor 1 receptor; CXCL8, C‐X‐C motif chemokine ligand 8 (interleukin 8); DAMPs, damage‐associated molecular patterns; DARC, Duffy antigen receptor for chemokines; ECs, endothelial cells; FBs, fibroblasts; HF, heart failure; HTRA3, HtrA serine peptidase 3; IGFBP7, insulin‐like growth factor‐binding protein‐7; NK, natural killer cells; OSM, cytokine Oncostatin M; PD‐1, programmed cell death protein 1; T, T cells; TGF‐β, transforming growth factor‐beta.

Immune cells play a pivotal role in coordinating responses in HF by contributing to both adaptive and maladaptive remodeling, and their activation is crucial for tissue repair and regeneration^[^ ^57^, ^58^ ^]^ Immune cell activation, transition, and regulation of other cardiac cells during the occurrence and progression of HF are summarized in **Figure** **4**. The cytokine oncostatin M (OSM) in pro‐inflammatory macrophages and programmed cell death protein 1 (PD‐1) in regulatory T cells, among others, were upregulated explicitly in key subpopulations after immune activation.^[^ ^59^ ^]^ However, different immune effector cells contribute disproportionately to each cardiovascular disease. Previous studies have summarized the mechanistic links between leukocyte subtype inflammation and HF in multiple cardiac disease remodeling. IL‐1 signaling promotes HF by impairing both systolic and diastolic functions. It affects systolic function by uncoupling L‐type calcium channels and adenylates cyclase from β‐adrenergic receptors, leading to desensitization of β‐agonists and affecting myocardial contractility. Diastolic dysfunction occurs due to the downregulation of phosphorylated proteins and sarcoplasmic reticulum calcium ATPase (SERCA), impairing Ca^2+^ reabsorption by the sarcoplasmic reticulum.^[^ ^57^ ^]^ Heterogeneous macrophage populations exert a significant influence on HF progression. The function of resident cardiac macrophages after myocardial injury showed heterogeneity; CCR2^+^‐resident cardiac macrophages exacerbated myocardial injury, promoted pro‐inflammatory leukocyte recruitment, and mediated dysfunction, whereas CCR2^‐^‐resident cardiac macrophages inhibited pro‐inflammatory leukocyte mobilization and protected the injured heart from adverse remodeling.^[^ ^60^ ^]^ Post‐injury cardiac repair critically relies on the efficient recruitment of immune cells, including monocytes and neutrophils, from both the spleen and circulating leukocytes. This process necessitates a transition of these cells from a pro‐inflammatory state toward a reparative program facilitated by the resolution of inflammation. Macrophages play a central role in this transition by synthesizing endogenous lipid mediators that promote timely tissue repair and prevent chronic inflammation, which can impair cardiac function. However, metabolic dysregulation can disrupt the inflammatory resolution process, leading to a block in cardiac repair. Lipid metabolism is intricately involved in this process, highlighting its crucial role in successful cardiac repair and regeneration.^[^ ^61^ ^]^ T cells, specifically CD8^+^ T cells, are essential in the adaptive response to HF caused by cardiac pressure overload. T cell depletion resulted in late‐stage heart protection and induced early‐stage HF by regulating macrophage gene expression, including AREG, OSM, and IGF1. The dynamic interplay between CD8^+^ T cells and macrophages is crucial for cardiac adaptation to stress, emphasizing that dynamic interactions between cardiac CD8^+^ T and macrophages are required for adaptation to cardiac stress based on scRNA‐seq study.^[^ ^62^ ^]^

Myocardial fibrosis, a hallmark of HF, arises from excessive immune cell activation and persistent inflammation. Single‐cell analysis of arrhythmogenic right ventricular cardiomyopathy (ARVC) patients reveals an accumulation of pro‐inflammatory macrophages and activated fibroblasts (FBs) in the right ventricle. These macrophages drive disease progression through interactions with FBs, and their activation is linked to genetic variants associated with cardiac arrhythmia. Notably, inhibiting the macrophage‐specific inflammasome NLRP3 significantly improves right ventricular function in an ARVC mouse model.^[^ ^63^ ^]^ FBs contribute to disease pathogenesis by driving both fibrosis and impaired angiogenesis. Combining scRNA‐seq with single T‐cell receptor sequencing technologies, identified AEBP1 as a critical regulator of myofibroblast fibrosis. Extensive infiltration of CD8^+^ T cells, CD4^+^ T cells, and CXCL8^hi^CCR2^+^ HLA‐DR^hi^ macrophages was detected in the fibrotic myocardium. These macrophages interacted with activated endothelial cells (ECs) via the duffy antigen receptor for chemokines (DARC) to promote leukocyte recruitment and infiltration.^[^ ^64^ ^]^ Rao et al. also confirmed that AEBP1 in ACTA2^+^ myofibroblasts regulates cardiac fibrosis. CCR2^+^ tissue‐intrinsic macrophages and blood monocyte‐infiltrated inflammatory macrophages are the primary sources of IL‐1β. The maintenance of CD8^+^ T cells in the failing heart is self‐proliferating and replenished by circulating T cells. ACKR1^+^‐ ECs^,^ a subpopulation of pro‐inflammatory ECs, was found to be enriched in failing hearts and promote inflammatory cell infiltration. IL34 from ACTA2^+^ myofibroblasts activated colony‐stimulating factor 1 receptor (CSF1R) on CCR2^+^ macrophages, stimulating CXCL8 secretion. CXCL8 subsequently induced ACKR1^+^‐ECs to upregulate inflammatory cell recruitment genes, amplifying the inflammatory response.^[^ ^65^ ^]^ Additionally, the scRNA‐seq study identified serine peptidase 3 (HTRA3) as a pivotal regulator of cardiac fibrosis and HF pathogenesis. Cardiac FBs were found to modulate the development of HF through the HTRA3‐TGF‐β‐IGFBP7 signaling axis. Specifically, pressure overload‐induced downregulation of HTRA3 expression in cardiac FBs led to the activation of transforming growth factor‐beta1 (TGF‐β1) signaling. This signaling cascade subsequently promoted cardiac fibrosis and HF progression by facilitating the accumulation of DNA damage within CMs.^[^ ^66^ ^]^ As described above, many studies based on scRNA‐seq have revealed the mechanism of immune cell imbalance and inflammation in promoting HF. Immune activation occurs in all immune cell types after myocardial remodeling far more cell types than previously thought. One study using spatial transcriptomics and scRNA‐seq maps the immune landscape post‐myocardial infarction (MI) and identifies TREM2‐expressing macrophages as a potential therapeutic avenue for HF.^[^ ^67^ ^]^ Integrating scRNA‐seq to explore macrophage heterogeneity, four genes (CD163, RNASE2, LYVE1, VSIG4) were identified as potential diagnostic markers for HF and developed a validated two‐gene diagnostic model, highlighting the role of macrophage infiltration in HF diagnosis.^[^ ^59^, ^68^ ^]^ Polyclonal, non‐antigen‐specific B cells rapidly infiltrate the infarcted myocardium via the CXCL13‐CXCR5 axis, contributing to local TGF‐β1 production without impacting cardiac function or morphology.^[^ ^69^ ^]^ Based on the existing scRNA‐seq and snRNA‐seq datasets from human hearts, revealing the presence of B cells within the human myocardium, integrated into a dynamic cellular network that exhibits disease‐specific alterations in cardiomyopathy, suggesting a potential role for naive myocardial B cells in the pathogenesis of dilated cardiomyopathy (DCM).^[^ ^70^ ^]^ In brief, immune cells of various types and locations, including resident and circulating cells, exhibit varying degrees of activation/inhibition during disease, and they are dynamic changes during the regulation of HF.

Meaningfully, SCS findings can guide clinical treatment or deepen understanding of clinical treatment plans. Some genes are detected and considered as therapeutic potential targets in HF treatment, such as ANGPTL4 for HFpEF,^[^ ^71^ ^]^ SPP1^+^ macrophages for AF treatment.^[^ ^72^ ^]^ For example, cytokine Osm is expressed both in proinflammatory cytokine‐producing M1 subclusters and cardiac patient hearts, which may offer a novel explanation for the failure of anti‐tumor necrosis factor therapy trials in patients with HF. PD‐1 expression at 1 week after TAC offers an explanation for recent reports of anti–PD‐1 treatment in cancer immunotherapy leading to fatal myocarditis.^[^ ^59^ ^]^

### 3.3 Tissue Ischemia: Severe Myocardial Ischemia Leading to HF

Severe myocardial ischemia and hypoxia, such as MI, may cause myocardial necrosis and decreased contractility, leading to HF. Normal adult rod CMs are homogeneous, with almost identical gene expression across mononuclear, binuclear, and multinuclear cells. In contrast, hypertrophic CMs showed significant alterations and marked heterogeneity, displaying characteristics of hypoxia‐induced responses. Specifically, NPPA, NPPB, HIF1α, EGR1, ACTA1, HES1, and ANKRD1, responsible for hypoxia response and muscle function, were identified as top DEGs between basal and CH states. The hypertrophic CMs revealed two distinct subpopulations based on HIF1α expression levels, with the HIF1α high cluster exhibiting more significant transcriptional activity, particularly in genes related to angiogenesis and glycolysis, highlighting the considerable impact of HIF1α on the hypertrophic CMs transcriptome and its role in adaptation to hypoxia. The hypoxic response of hypertrophic CMs drives differential vascular growth, which is the main reason for the heterogeneity of CMs in the remodeled hypertrophic heart.^[^ ^73^ ^]^ The expression of NPPA and NPPB genes increased in CMs during CH.

Previous studies using scRNA‐seq have suggested that PRKAR1A and SDCBP can be potential predictors and are involved in regulating the mechanism of HF after acute MI through inflammation, apoptosis, and angiogenesis.^[^ ^74^ ^]^ Farbehi et al. identified 30 subpopulations from 9 cell types by scRNA‐seq of mouse heart tissue on days 3 and 7 post‐infarction. Analysis of cell trajectories identified an unreported fibroblast characterized by high expression of the anti‐WNT signaling pathway, while three fibroblast subtypes were newly defined, expressing pro‐ or anti‐fibrogenic signaling, respectively.^[^ ^75^ ^]^ These results suggest that FBs mediate the mechanism of severe myocardial ischemia that leads to decreased cardiac function and HF, providing insight for in‐depth analysis of the cardiac composition and pathological processes in extreme ischemic states. The bulk RNA‐seq, scRNA‐seq, and scATAC‐seq (single‐cell assay for transposase‐accessible chromatin with sequencing) integrated methodology identified a subset of activated FBs exhibiting observable pro‐fibrotic characteristics that manifested following MI in mice. Remarkably, these FBs displayed a notable increase in the expression of CTHRC1 within the scar tissue, providing further evidence of its involvement in the fibrotic response associated with MI. CTHRC1 is involved in collagen matrix synthesis through the TGF‐β signaling pathway. Therefore, CTHRC1 may be a potential intervention target for ventricular remodeling and myocardial fibrosis after MI.^[^ ^76^ ^]^ Function changes of ECs due to infarction ischemia, resulting in significant heterogeneity. However, adapting ECs to post‐MI injury is vital in the angiogenic response to tissue repair after MI. Tombor et al. performed scRNA‐seq on the non‐cardiomyocytes (NCMs) of the mouse heart at 0, 1, 3, 5, 7, 14, and 28 days after MI and described in detail the characteristics of cellular gene expression at different periods after MI. Five different ECs subsets appeared after MI. The endothelial‐mesenchymal transition may be reversible due to hypoxic and inflammatory injury.^[^ ^77^ ^]^ Recently, a study investigated the role of cuproptosis in the progression of ischemic HF. Cuproptosis is a new programmed cell death pattern discovered by Todd R. Golub and Peter Tsvetkov group in 2022. Copper affects the TCA cycle by disrupting the lipoylation of pyruvate dehydrogenase complex components. This study conducted a comprehensive evaluation of the association between cuproptosis and the immune microenvironment in ischemic HF, encompassing infiltrated immunocytes, immune reaction gene‐sets and human leukocyte antigen genes. Moreover, they identified two different cuproptosis‐mediated expression patterns in ischemic HF and explored the immune characteristics associated with each pattern.^[^ ^78^ ^]^

### 3.4 Extracellular Matrix Remodeling

Extracellular matrix (ECM) remodeling is a crucial pathological aspect of HF, causing ongoing myocardial damage during both systolic and diastolic heart phases.^[^ ^79^ ^]^ ECM, produced and modified by myocardial cells (mainly cardiac FBs), also facilitates communication among all cardiac cells.^[^ ^80^ ^]^ SCS aids in understanding the interaction between ECM and cellular communication.^[^ ^81^ ^]^ HCM scRNA‐seq and snRNA‐seq datasets showed up‐regulation of ECM genes like LUM, DCN, FN1, CTGF, and COLIA2 in HCM cardiomyocytes, indicating their role in ECM remodeling.^[^ ^85^ ^]^ ECM of HCM includes fibrous collagen, interstitial proteoglycans, SLRP (small leucine rich proteoglycan) family members, and the main CSPG (chondroitin sulfate proteoglycan), versican. Versican and other proteoglycans accumulate in the scarred myocardium of patients with ischemic HF.^[^ ^83^ ^]^ Versican, a cardiac fibroblast‐derived pro‐proliferative proteoglycan, is cleaved by a disintegrin and metalloproteinase with thrombospondin motifs (ADAMTS) proteases during cardiac remodeling.^[^ ^84^ ^]^ ADAMTS5 as one of the most abundant proteases secreted by mouse cardiac FBs regulates the accumulation of the proteoglycan versican.^[^ ^79^ ^]^ However, the FB subsets enriched in ADAMTS5 are the least enriched for versican.^[^ ^85^ ^]^ ADAMTS5 is the main population of ventricular FBs, different from FBs in the atrium. These findings implicate an individualized treatment in ischemic HF, such as modulating ECM remodeling using β‐blockers to reduce versican accumulation but no affecting ADAMTS5.^[^ ^79^ ^]^

FBs play a key role in cardiac ECM remodeling and CMs dedifferentiation.^[^ ^73^, ^75^, ^85^ ^]^ All the genes involved in ECM organization and collagen biogenesis/formation/degradation, including LTBP2, LTBP1, COL3A1, MFAP4, COL12A1, COL1A1, COL1A2, MMP2, TIMP2, and PCOLCE2, are upregulated in the HF samples and are specifically secreted by FBs.^[^ ^86^ ^]^ Resident FBs synthesize ECM after MI to form fibrosis, leading to cardiac dysfunction and HF.^[^ ^87^ ^]^ ScRNA‐seq demonstrated that resident FBs consisted of 7 subclusters, in which the profibrotic FB population increased under chronic MI.^[^ ^88^ ^]^ Different FB subcluster displays different genes. For example, cluster FB‐S3 enriches expression of fibrosis‐associated genes NOX4 and IGF1, and cluster FB‐S4 exhibits clear upregulation of pro‐fibrotic markers, including ADAMTS4 (encoding a pro‐fibrotic metalloprotease), VCAN (encoding the proteoglycan versican), and AXL (encoding a receptor tyrosine kinase associated with pathologic remodeling).^[^ ^89^ ^]^ The snRNA‐seq of patients with hypertrophy and HF also detected 7 distinct fibroblast clusters, and one of subclusters was characterized by high expression of POSTN, RUNX1, CILP, and a target gene adipocyte enhancer‐binding protein 1 (AEBP1) in patients with established cardiac hypertrophy and HF.^[^ ^90^ ^]^ Cilp^+^ fibroblast and Thbs4^+^ fibroblast are key contributors to ECM remodeling.^[^ ^91^ ^]^ Another study confirms that Thbs4 expression is upregulated after injury and drives cardiac fibrosis, while the transcription factor TEAD1 may be a regulator of fibroblast activation.^[^ ^92^ ^]^ Cardiac reprogramming suppressed fibroblastic gene expression in chronic MI by means of conversion of profibrotic FBs to a quiescent antifibrotic state.^[^ ^88^ ^]^ Cytoskeleton‐associated protein 4 as a new marker for activated FBs during ischemic injury that appears to attenuate myofibroblast activation.^[^ ^93^ ^]^ All these results reveal that SCS can explain the transcriptional switch toward a pro‐fibrotic ECM composition in MI, cardiac hypertrophy and HF.

Importantly, FBs offer potential targets for therapeutic intervention and biomarkers for HF. Knocking down the p53 expression of FBs can lower levels of genes encoding important ECM proteins.^[^ ^94^ ^]^ Through scRNA‐seq detection of non‐myocardial cells in the injured and uninjured areas of the heart after post‐MI, it was found that Col5a1 deficiency leads to an increase in the number of myofibroblasts in the scar tissue of the heart, as well as an increase in the expression of myofibroblast markers and other ECM genes, resulting in an increase in scar size.^[^ ^95^ ^]^ A unique FB subpopulation expressing a high level of Cthrc1 (collagen triple helix repeat containing 1) exhibits a clear profibrotic signature and localizes into the scar after MI in mice.^[^ ^76^ ^]^ In humans, exposure to calcitonin resulted in extensive changes in atrial fibroblast translation levels, including ECM proteins and pathways associated with fiber formation, infection and immune response, and transcriptional regulation. Calcitonin derived from human cardiac FBs controls fibroblast proliferation and bone morphogenetic protein 1 (BMP1)‐related collagen processing by binding to calcitonin receptors on the cardiac fibroblast membrane. Disruption of calcitonin‐calcitonin receptors leads to excessive atrial fiber formation and promotes arrhythmia.^[^ ^96^ ^]^

In addition to FBs, many other cell types in the cardiac cellulome are also involved in promoting ECM remodeling, such as B cells, CMs, dendritic‐like cells, ECs, endocardial cells, granulocytes, pericytes, and T cells.^[^ ^91^ ^]^ The activity of macrophages in ICM seems to be higher than in NF and DCM. Upregulation of CCL3/CCL4‐CCR5 by autocrine ligand receptors in ICM was also observed in the validation dataset. Consistently, the expression of CCL3 and CCL4 in ICM macrophages was higher than that in DCM and normal hearts. These results further indicate that activation of chemokine signaling in macrophages may induce cardiac fibrosis during HF.^[^ ^86^ ^]^ A scRNA‐seq analysis of wound border zone cells in zebrafish hearts showed that mac4^+^ macrophages were involved in “collagen degradation” (mmp2, mmp14a, and mmp14b), and “collagen biosynthesis and modifying enzymes” (col12a1a, pcolce2b, col4a2, and col6a3).^[^ ^97^ ^]^ These results reveal that special macrophages are important for remodeling and degradation of ECM, which contributes to CM invasion into the injured cardiac tissue during regeneration.

In addition, accompanied by transcriptional alterations associated with hypoxia, angiogenesis, and migration, ECs exhibit a proliferative response after an injury that does not persist during later remodeling.^[^ ^92^ ^]^ SOX9‐induced ECM, growth factor, and inflammatory gene expression lead to matrix deposition by ECs. Moreover, mouse ECs activated neighboring FBs that then migrated and deposited matrix in response to SOX9, a process partly mediated by the secreted growth factor CCN2, a direct SOX9 target; endothelial cell‐specific Sox9 deletion reversed these changes. These findings suggest a role for endothelial SOX9 as a fibrosis‐promoting factor in different mouse organs during disease and imply that ECs are an important regulator of fibrosis.^[^ ^98^ ^]^ Paracrine factors such as TGF‐β1 and fibroblast growth factors are expressed by epicardial‐derived cardiac FBs and ECs, leading to epithelial‐mesenchymal transition and contributing to the development of myocardial fibrosis, apoptosis, arrhythmia, and cardiac dysfunction.^[^ ^99^ ^]^

### 3.5 Small Molecular Mediators

**Figure 5:**
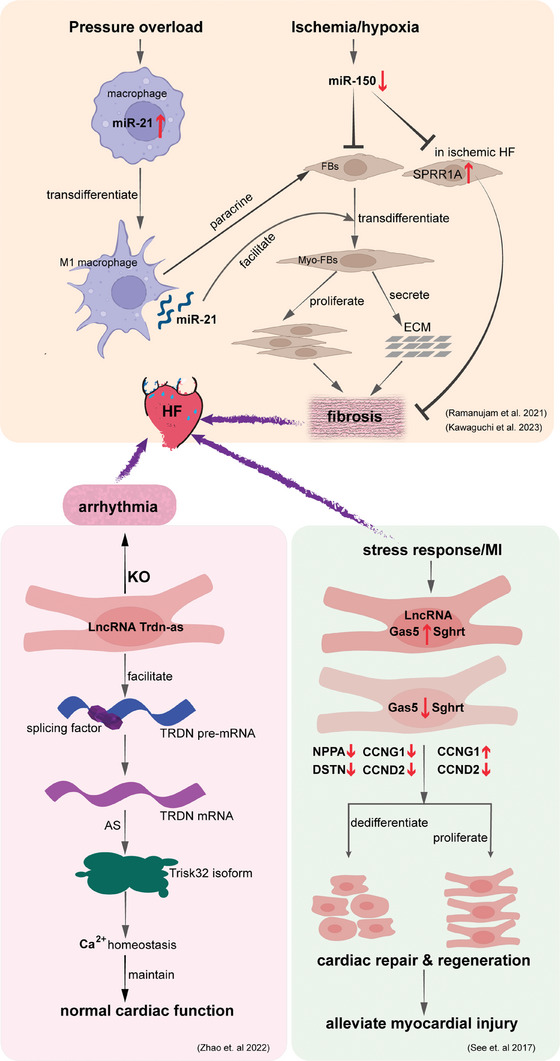
Small molecular mediators in heart failure indicated by SCS. Pressure overload induces miR‐21 increase in cardiac macrophage, which can activate FBs through paracrine. But ischania/hypoxia induces miR‐51 decrease, inhibiting FBs transformation. Both of them can induce fibrosis and promote HF progress. MiR‐51 also upregulates SPRR1A in ischemic HF, which can suppress fibrosis. LncRNA also are involved in HF. Knockout of *Trdn‐as* damages the recruitment of splicing factors to *Trdn* pre‐mRNA, causing abnormal composition of triadin isoforms in the heart, Ca^2+^ mishandling, and susceptibility to cardiac arrhythmias. LncRNAs *Gas5* and Sghrt in FBs are upregulated after stress response or MI, regulating co‐expressed genes within the same gene regulatory network including cell cycle genes: *CCNG1* and *CCND2* and others: *NPPA* and *DSTN*. AS, alternative splice; ECM, extracellular matrix; HF, heart failure; KO, Knockout; FBs, fibroblasts; MI, myocardial infarction; lncRNA, long noncoding RNA; *Trdn‐as*, a CM‐specific lncRNA encoded by the antisense strand of the *triadin* gene.

Failing myocardium was characterized by prominent increases in macrophage and fibroblast numbers.^[^ ^100^ ^]^ It is generally assumed that microRNA‐21 (miR‐21) exerts its profibrotic action primarily in FBs of the failing heart.^[^ ^101^ ^]^ However, another study found that miR‐21, as the single highest expressed microRNA in cardiac macrophage, primarily determined macrophage‐fibroblast communication through promoting the transition from quiescent FBs to myofibroblasts.^[^ ^102^ ^]^ As shown in **Figure** **5**, the inhibition of miR‐21 can reverse the increase of macrophage and fibroblast numbers in failing myocardium.^[^ ^100^ ^]^ Thus, miR‐21 as a target is an effective antifibrotic strategy in HF.

FBs and myofibroblasts represent potential targets in fibrotic cardiac disorders.^[^ ^103^ ^]^ However, the lack of genes uniquely expressed in these 2 cell types has precluded the development of specific therapies. Several studies have evaluated cardiac nonmyocyte populations by analysis protein‐coding genes in single‐cell approaches.^[^ ^75^, ^91^, ^93^ ^]^ Compared with protein‐coding genes, long non‐coding RNAs (lncRNAs) are more cell‐specific. So, this method is well‐suited to assess the seemingly homogenous cell populations in scRNA‐seq experiments, such as FBs and myofibroblasts. Aghagolzadeh et al. investigated the lncRNA transcriptome's potential in characterizing cardiac cell populations after post‐MI to identify heterogeneity within fibroblast and myofibroblast populations and uncover subpopulation‐specific markers as potential therapeutic targets for heart disease. Their findings underscored the utility of lncRNA expression in distinguishing cardiac cell types. They identified a novel myofibroblast‐specific lncRNA, fibrogenic LOX‐locus enhancer RNA (FIXER), thereby highlighting its promise as a therapeutic target for cardiac fibrosis.^[^ ^103^ ^]^ SCS in peripheral blood mononuclear cells revealed a novel human‐specific lncRNA called HEAT4 (heart failure‐associated transcript 4) associated with HF, acute MI, or cardiogenic shock. Blood HEAT4 is primarily secreted by anti‐inflammatory CD16^+^ monocytes in patients with HF. Increased HEAT4 levels result in a shift toward more CD16^+^ monocytes. HEAT4 activates anti‐inflammatory and inhibits proinflammatory gene expression through binding to S100A9 to cause a monocyte subtype switch, thereby reducing inflammation.^[^ ^104^ ^]^ Actually, a large number of lncRNAs in nuclei of CMs were discovered using snRNA‐seq. In these lncRNAs, Gas5 and Sghrt are highly expressed in TAC CMs and are considered as key nodal hubs to regulate co‐expressed genes within the same gene regulatory network including cell cycle genes and others (Figure 5).^[^ ^13^ ^]^ To conclude, lncRNAs as potential targets intervene in HF.

Combining scRNA‐Seq with other technologies, such as scATAC‐seq and miRNA‐seq, yields more well‐rounded and multi‐dimensional insights and reveals the novel molecular mechanisms of HF. The combining miRNA‐seq with scRNA‐seq analysis found that a subset of DEGs in various cell types were among the predicted targets of the up‐regulated miRNAs and most of the identified targets were under the control of mmu‐miR‐3473a and mmu‐miR‐1983, suggesting that these two miRNAs may be the master regulators of HF development. Analysis of scATAC‐seq revealed a NO biosynthesis‐related gene regulation pattern in ECs of failing hearts.^[^ ^105^ ^]^

## 4 The Potential Help of SCS Technology for Clinical

### 4.1 Inter‐Cellular Communication in the Heart as a Possible Therapeutic Target for HF

**Figure 6:**
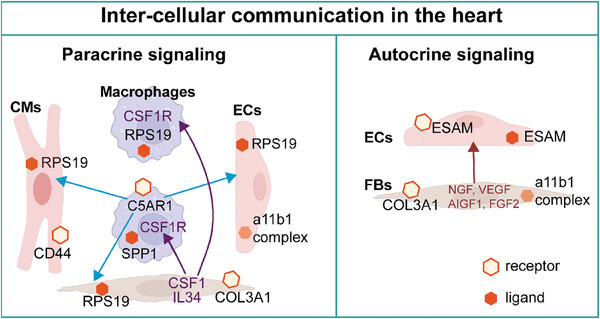
Inter‐cellular communication in the heart as a possible therapeutic target for HF. Extensive cellular interactions between different types of cardiac cells play an important role in HF. Inter‐cellular communication in the heart mainly includes paracrine signaling and autocrine signaling. Macrophages communicate with CMs, ECs, and FBs through receptor‐ligand interaction, such as C5AR1 in macrophages as a receptor interacting with RPS19 in CMs, ECs, and FBs as a ligand for paracrine signaling. FBs express CSF1 and IL34, which regulate macrophage growth and survival through CSF1R signaling. FBs also express growth factors, including NGF, VEGFA, IGF1, and FGF2, which are vital for supporting the development of neurons in the autonomic nervous system, ECs, and parietal cells. CMs, cardiomyocytes; ECs, endothelial cells; ESAM, endothelial cell‐selective adhesion molecule; FBs, fibroblasts; NGF, nerve growth factor; VEGFA, vascular endothelial growth factor A; IGF1, insulin‐like growth factor‐1; FGF2, fibroblast growth factor 2.

Cell‐specific transcriptional signatures associated with HF in patients showed CMs converged to common disease‐associated cellular states, while FBs and myeloid cells underwent significant diversification. ECs and pericytes showed global transcriptional changes without changes in cellular complexity.^[^ ^106^ ^]^ An snRNA‐seq of nearly 600 000 nuclei in human hearts revealed extensive cell type alterations in dilated and hypertrophic cardiomyopathy (HCM) hearts. In DCM and HCM hearts, CMs statistically decrease, but vascular smooth muscle cells increase, and FBs are activated. Additionally, macrophages and lymphocytes increase in DCM hearts, whereas lymphatic ECs increase in HCM hearts.^[^ ^107^ ^]^ In these cell types, NCM cell types are the major cellular communication hubs in specific regions and actively regulate CM behavior using scRNA‐seq.^[^ ^108^ ^]^ NCMs, such as FBs, offer potential targets for therapeutic intervention and biomarkers for HF.^[^ ^107^ ^]^ Clarifying the interaction network of cardiac cells is necessary to understand cardiac homeostasis and disease. C5AR1 in macrophages served as a receptor, and RPS19 in CMs, ECs, and FBs as a ligand for paracrine signaling.^[^ ^109^ ^]^ Skelly found that all CMs exhibit a robust intercellular communication network, with FBs showing the highest level of communicative capacity. Notably, FBs express CSF1 and IL34, which regulate macrophage growth and survival through CSF1R signaling. FBs also express growth factors, including NGF, VEGFA, IGF1, and FGF2, which are vital for supporting the development of neurons in the autonomic nervous system, ECs, and parietal cells. Furthermore, a complex interconnection exists between CMs, FBs, ECs, macrophages, smooth muscle cells, Schwann cells, NK cells, B cells, and T cells.^[^ ^110^ ^]^ A comprehensive analysis of cellular composition and expression profiles at the single‐cell level in the adult heart highlights the heterogeneity of CMs, pericytes, and FBs, highlighting the collaborative network between cardiac FBs, macrophages, and CMs in the atrium and ventricle.^[^ ^85^ ^]^ Wu et al. demonstrated the cellular diversity of the left ventricle while revealing the extensive intercellular communication network and found that autocrine and paracrine signaling underlies LV homeostasis.^[^ ^109^ ^]^ Extensive cellular interactions between different types of cardiac cells play an important role in HF, as Wang et al.^[^ ^108^ ^]^ reported. These findings provide insight into the role of intercellular communication in HF, new avenues for the study of cardiac cells, and a resource for deeper investigation of cell type‐specific transcriptional networks in cardiac homeostasis with HF (**Figure** **6**).

### 4.2 SCS Technology Revealing the Composition, Transformation and Interaction of Cell Groups in the HF Microenvironment

The HF microenvironment is characterized by local inflammation, hypoxia, oxidative stress, impaired lymphatic drainage, and impaired recovery of cardiac contractibility. Ongoing inflammation and endothelial dysfunction occurs within the local microenvironment of HF.^[^ ^111^ ^]^ SCS is a powerful tool for exploring the complex HF microenvironment, further promoting personalized treatment.

ECs are the most abundant cell type of cardiac interstitial cells and act as a sound barrier between circulating blood and CMs.^[^ ^112^ ^]^ ECs affect myocardial fibrosis mainly through regulation of endothelial‐to‐mesenchymal transition (EndMT) and paracrine signaling pathways, such as SIRT1 activated by resveratrol attenuated cardiac fibrosis by regulating EndMT via the TGF‐β/SMAD2/3 pathway.^[^ ^113^ ^]^ A recent study found that ECs directly contribute to fibrosis in the heart by activating the transcription factor SOX9, producing ECM components and activating neighbouring FBs.^[^ ^98^ ^]^ Deleting ETS‐1 in ECs can reduce angiotensin II‐induced cardiac fibrosis by suppressing EndMT.^[^ ^114^ ^]^ ACKR1^+^‐Ecs acted as the hub in cell‐cell interactions.^[^ ^108^ ^]^ The critical role of FBs in HF and cardiac fibrosis has already been described, and they also induce ECs inflammation and dysfunction in a paracrine manner.

Interestingly, stem cells can remodel the HF pathological microenvironment. Profound changes in circulating immune cells were found in HF patients. Using scRNA‐seq of murine and human heart tissue, researchers characterized the cellular landscape during heart hypertrophy, focusing on CD34^+^ cells. The non‐bone marrow‐derived CD34^+^ cells differentiate into FBs and ECs, contributing to cardiac fibrosis, while bone marrow‐derived CD34^+^ cells produce immune cells only under pressure overload. Depletion of CD34^+^ cells in a mouse model alleviated fibrosis and improved heart function. Further single‐cell pseudotime analysis, combined with in vitro cell culture studies, revealed the involvement of WNT‐β‐catenin and TGFβ1/SMAD pathways in regulating CD34^+^ cell differentiation into FBs.^[^ ^115^ ^]^ Another SCS study of mouse and human hearts reveals that a population of non‐bone marrow‐derived Sca1^+^ cells differentiate into FBs during cardiac hypertrophy. Depletion of these Sca1^+^ cells mitigate fibrosis and improves cardiac function in mice. Mechanistically, these cells appear to contribute to fibrosis through the Wnt4‐PDGFRα pathway, suggesting a potential therapeutic avenue for HF.^[^ ^116^ ^]^ These findings highlight the role of CD34^+^ cells and Sca1^+^ cells in cardiac fibrosis and suggest their potential therapeutic avenues for HF treatment.

**Figure 7:**
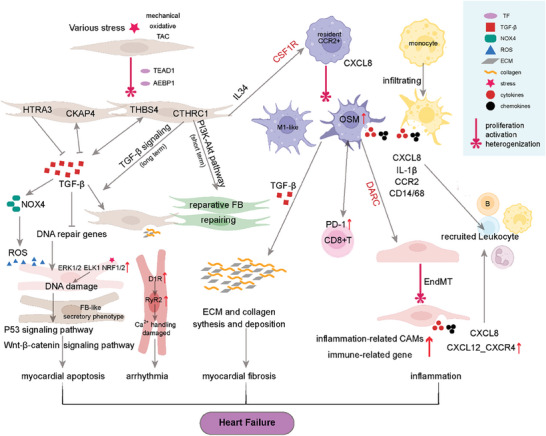
From cell to gene: multiple mechanisms work together to mediate HF. Cell composition and cell types undergo extensive changes during HF. ECs, FBs, and immune cells all contribute to the occurrence of HF. ECs affect myocardial fibrosis mainly through regulating EndMT and paracrine signaling pathways, such as SIRT1 activated by resveratrol attenuated cardiac fibrosis by regulating EndMT via the TGF‐β/Smad2/3 pathway. FBs in HF and cardiac fibrosis also induced ECs inflammation and dysfunction in a paracrine manner. Immune cells, such as macrophages, T cells, neutrophils, B cells, NK cells, and mast cells, are extensively activated after pressure overload and are involved in HF progression. AEBP1, AE Binding Protein 1; B, B cells; CAMs, cell adhesion molecule; CCR2, CC chemokine receptor 2; CKAP4, cytoskeleton‐associated protein 4; CSF1R, colony stimulating factor 1 receptor; D1R, dopamine D1 receptor; ECM, extracellular matrix; EndMT, endothelial‐to‐mesenchymal transition; FBs, fibroblasts; HTRA3, HtrA serine peptidase 3; M1‐like, M1‐like macrophage; NOX4, NADPH oxidase 4; OSM, cytokine oncostatin M; PD‐1, programmed cell death protein 1; RyR2, ryanodine receptor 2; ROS, reactive oxygen species; TAC, transverse aortic constriction; TEAD1, TEA domain transcription factor 1; TGF‐β, transforming growth factor‐beta; TF, transcription factor.

The protein structures acting as mechanotransducers are the first step in the process that drives physiological and pathological hypertrophy, remodeling of the heart, and the transition to HF.^[^ ^117^ ^]^ Multiple genes and pathways lead to myocardial cell apoptosis and death, causing HF. Overexpression of miR‐125b can inhibit CMs apoptosis by targeting BAK1, thereby alleviating cardiac function damage in mice with HF.^[^ ^118^ ^]^ The ROS‐ATM‐LARP7‐SIRT1‐OXPHOS pathway is essential to maintaining normal heart function. The LARP7 gene is critical in regulating mitochondrial biogenesis, energy production, and cardiac function by modulating SIRT1 activity. Reduced LARP7 levels due to activation of the ATM pathway may contribute to the development of HF, while LARP7 reconstitution in injured hearts may provide myocardial protection.^[^ ^119^ ^]^ Inflammatory factors S100A8 and S100A9 are involved in autophagy and apoptosis and can regulate these processes in CMs via the MAPK or PI3K‐AKT signaling pathways.^[^ ^120^ ^]^ In summary, during HF, the whole heart's cellular composition and cell types undergo extensive changes, which scRNA‐seq can detect. CMs undergo apoptosis or death due to pressure overload, energy metabolism disorder, or ionic remodeling, and NCMs are widely involved in regulating this process. The EndMT, the activation and proliferation of FBs, the activation of immune cells, and the type change all contribute to decreased cardiac function and the occurrence of HF under the regulation of the expression of specific genes at certain moments and central differential genes in CMs, FBs, and ECs shown as in Table S1 (Supporting Information). Gene expression changes often accompany changes at the cell level, and conversely, the differential expression of a series of genes regulates the changes in the cell in the whole process, and the scRNA‐seq establishes a technical foundation (**Figure** **7**).

In summary, scRNA seq helps to explore the complexity of cell populations in the HF microenvironment and reveals different cell subpopulations with the potential to become personalized targets, such as immunotherapy and stem cell therapy.

### 4.3 Precision Medicine

In addition to deepening our understanding of HF pathomechanisms, SCS technology can help doctors develop personalized therapies for different types of HF. HFpEF treatment is the most unsatisfactory in cardiovascular medicine. Currently, there is still no specific treatment for HFpEF. The SGLT2 inhibitor dapagliflozin has potential therapeutic benefits for HFpEF due to its anti‐inflammatory effects.^[^ ^53^ ^]^ With the help of scRNA‐seq, researchers found that dapagliflozin's cardioprotective effects in HF are independent of SGLT2 inhibition and are primarily mediated through the suppression of macrophage‐driven inflammation and subsequent fibroblast activation.^[^ ^121^ ^]^ A snRNA‐seq of failing and non‐failing human hearts helped identify unique cellular compositions and transcriptional profiles. These findings highlight the influence of pathogenic genetic variants on HF mechanisms and suggest that specific molecular and cellular contexts in cardiomyopathies could inform tailored therapeutic approaches, which provides a foundation for future interventions that might enhance personalized medicine for cardiomyopathies and HF.^[^ ^122^ ^]^

A large cell therapy trial on patients with chronic HF with low ejection fraction indicates that the effectiveness of cell therapy has a synergistic and additional effect with state‐of‐the‐art HF drugs.^[^ ^123^ ^]^ This study indicates that cell therapy provides potential options for the treatment of HF. With the help of SCS, more and more cells have been proven to be potential target cells for HF, such as CD34^+^ cells,^[^ ^115^ ^]^ Sca1^+^ cells,^[^ ^116^ ^]^ Treg^+^ and Th17^+^ T cells.^[^ ^85^ ^]^ Another study uncovered that active engagement of NCMs by injection of ACKR1^+^ ECs can preserve cardiac function after injury by regulating the behavior of CMs.^[^ ^108^ ^]^ These studies confirm that cell‐type‐targeted intervention of HF is effective and feasible.

It is worth emphasizing that peripheral blood is easier to obtain than myocardial tissue in clinics. Therefore, SCS is a good choice to detect the dynamic alterations of peripheral blood mononuclear cells during HF for providing personalized treatment plans for HF patients. Recently, peripheral blood samples from 4 HF patients before and after mesenchymal stem cell (MSC) therapy were performed scRNA‐seq. This study identifies treatment‐responsive CD14^+^ monocytes as a crucial biomarker for assessing the suitability of MSC therapy and determining which HF patients could benefit from it.^[^ ^124^ ^]^

## 5 Challenges and Current Status of scRNA‐Seq in the Heart

The scRNA‐seq offers new tools and technical foundations for decoding the complex structure and pathological mechanisms of the heart, but it also poses significant challenges and thus limits the development of cardiac single‐cell studies, especially in single‐cell sample preparation.

### 5.1 Single‐Cell Sample Preparation

Myocardial cell discs have a concave and convex cell membrane topology and undergo specialized differentiation to form desmosomes. The arrangement and distribution of ECM components, such as collagen, elastin, adhesion, and glycoprotein molecules, create a complex network in the myocardium. Additionally, CMs are highly sensitive to oxygen deprivation, leading to significant structural changes and cell death if hypoxia persists.^[^ ^125^, ^126^ ^]^ Based on the properties of the heart above, several challenges must be addressed when preparing the single‐cell suspension. First, cardiac myocytes' heterogeneity in size and properties makes their dissociation difficult. The enzymatic elimination technique yields low cell numbers, while the perfusion method necessitates access to vascularized tissue, limiting sampling possibilities. Second, CMs are too large, resulting in low compatibility with existing high‐throughput single‐cell platforms such as microfluidic and droplet systems. Adult CMs are elongated in shape, with an average cell body diameter greater than 100 µm and more prolonged and larger in some pathological states. Mainstream SCS platforms such as 10 × Genomics and Drop‐seq are more suitable for cells smaller than 30 µm in diameter. Third, the NCMs are minor and hard to digest, which requires more stringent dissociation conditions, and the process will further damage the larger CMs. Finally, when obtaining specific pathological cardiac cells, the rigid fiber scar caused by cardiac remodeling is brutal to dissociate into single cells, which adds to the difficulty of obtaining single pathological cells.

### 5.2 Limitations in snRNA‐Seq and Coverage

With the development of technology, the ICELL8, based on microfluidic chip technology, offers new possibilities for CM capture regardless of cell size, with the advantage of high throughput and low cost. A large particle FACS (lp‐FACS) with a channel size of 500 µm can successfully isolate adult mouse CMs with good cell morphology and high RNA quality.^[^ ^127^ ^]^ In addition, snRNA‐seq techniques are commonly used to detect clinical samples with long sampling times and difficulty in sampling, as well as samples with large size and difficulty in capturing, such as CMs, taking advantage of the nucleus's non‐degradation during repeated freeze‐thaw processes.^[^ ^13^, ^128^ ^]^ It has been used in many studies (Table S2, Supporting Information), such as single‐nucleus profiling of human DCM and HCM, and dynamic transcriptional responses to injury in regenerative and non‐regenerative CMs.^[^ ^107^, ^129^ ^]^ However, the snRNA‐seq has certain limitations. Different genes are not uniformly localized within the cell; mRNA is thought to predominantly reside in the cytoplasm, while some are more localized in the nucleus.^[^ ^130^ ^]^ When only snRNA‐seq is used for analysis, nuclear‐enriched transcripts may overemphasize biological processes such as cell cycle, transcriptional, and ubiquitous cycles, resulting in distortion of the relative abundance of gene transcripts, affecting cell type identification and cell clustering.^[^ ^131^, ^132^ ^]^ Therefore, multidimensional and multifaceted techniques and methods should be combined for specific tissue samples to complement and corroborate each other. More suitable single‐cell capture tools for cardiac tissues will provide better assurance for studies related to heart diseases.

Besides the inherent challenges in obtaining individual cardiac cell samples, scRNA‐seq studies present many complexities and obstacles in cardiac research. These challenges include the inability of sequencing technology to capture gene dynamics in complex biological processes simultaneously and the transient and low‐abundance populations that may be present. One of the current significant scRNA‐seq limitations in cardiac research is its limited coverage, which is expected to capture only 5%–20% of the complete transcriptome for each cell.^[^ ^110^, ^133^, ^134^ ^]^ This limited coverage impairs a thorough understanding of cell function and identity. With myocardial cells composing ≈30% of the cardiac tissue, more extensive genomic coverage is required to ascertain their similarity. The substantial representation of mitochondrial genes in myocardial cells can attenuate transcriptional levels, reducing genome coverage. Consequently, the obtained information primarily concerns the most prevalent genes, making it challenging to identify rare subtypes within cell populations characterized by low‐expression genes. Additionally, it remains possible that other cells might be inadvertently included during the cell sorting process, either within the sample itself or the sampling area, further complicating the identification of these rare populations. Due to these different cellular biases in sorting strategies, scRNA‐seq technology cannot reliably capture the relative abundance of all adult cardiac cell types unbiasedly. To obtain a reliable and comprehensive transcriptomic landscape of the adult CMs, detection methods with higher sensitivity and coverage that require high numbers of cells must be developed.

### 5.3 Data Analysis

In terms of data analysis, the previous studies revealed remarkable heterogeneity in the expression of established CMs markers between individual subclusters of adult CMs, suggesting that marker genes are not exclusive to a specific cell type and mean potential contamination of gene expression among various cell types.^[^ ^89^, ^93^, ^135^ ^]^ Although we often calculate and verify the SCS data using different algorithms, the wet experiment is always the most accurate verification method. Apart from increasing the sampling cell numbers and verification by experiment, more specific markers and sorting methods must be developed.

When acquiring the data, the scRNA‐seq often encounters many observed zeros, indicating that certain genes in specific cells lack unique molecular identifiers or reads mapping to them. Zero values can represent undetected but expressed genes due to factors such as sequencing technology and cell sorting or biologically‐true absence of expression, which are challenging to distinguish.^[^ ^136^ ^]^ The primary methods, including model‐based, data smoothing, data reconstruction, and imputation with an external dataset or reference, and use for transfer learning, have been summarized by Lähnemann et al., and new approaches are constantly evolving to handle missing data in scRNA‐seq data.^[^ ^137^, ^138^ ^]^ However, circularity arises when imputation is based solely on the information in the imputed data set, which can lead to exaggerated correlations between genes/cells, amplifying the signal in the data and causing false positives in downstream analyses.^[^ ^139^ ^]^

### 5.4 Others

Transcription in organisms is a dynamic and uncertain process compared to the stability of the genome. Many scRNA‐seq studies use samples from a specific time point, leading to the potential loss of transiently expressed genes or cell‐type changes. The variability in analysis platforms can result in uncertainties in quantifying unknown cell types and states. Additionally, uncertainties in cell‐to‐gene quantification persist throughout sample preparation, sequencing, and data analysis. Maintaining accuracy in quantification and minimizing data bias are vital challenges in scRNA‐seq. DEGs are typically identified based on known cell types or experimental conditions, potentially overlooking cellular heterogeneity. Overcoming these challenges necessitates larger datasets encompassing diverse cell types and integrating multiple omics data for a comprehensive reference. While progress in building platforms based on other omics data is ongoing, integrating data across platforms and technologies remains a substantial hurdle.

## 6 Discussion and Perspective

HF is a complex pathological state characterized by reduced cardiac function and multifactorial mechanisms. At the molecular and cellular levels, CMs are subjected to a variety of damaging factors, leading to disruptions in cell signaling pathways. Energy metabolism disorders also play a crucial role in the progression of HF, contributing to myocardial cell damage and remodeling. The intricate interplay between these factors culminates in irreversible ventricular dysfunction, culminating in the onset and exacerbation of HF.

The advent of single‐cell omics technology represents a significant advancement in our ability to study HF. Unlike traditional bulk sequencing methods, which offer an aggregate view of genes expression, SCS enables the analysis of individual cells, revealing the heterogeneity and functional diversity that are fundamental to heart health and disease. This technological innovation has opened new avenues for exploring the molecular mechanisms underlying HF, identifying potential therapeutic targets, and personalizing medical interventions.

Recent developments in SCS have underscored its relevance to HF research. Novel methodologies and findings, particularly those emerging within the past years, have expanded our understanding of the cellular and molecular processes involved in HF. For example, the ability to profile individual cell transcriptomes has unveiled previously unappreciated cell populations and their distinctive roles in HF pathogenesis. The clinical implications of SCS studies are far‐reaching. By examining the molecular underpinnings of HF at the single‐cell level, researchers can pinpoint potential therapeutic targets and predict patient responses to various interventions. This approach facilitates the development of personalized medicine strategies, where treatments are customized to the unique genetic and cellular characteristics of each patient. Despite the promise of SCS, challenges in data analysis, integration, and interpretation remain. The complexity of single‐cell data demands advanced computational tools for effective processing and understanding. Future research efforts must focus on addressing these challenges to maximize the utility of SCS in HF research.

A comparison of SCS with traditional bulk sequencing methods provides a clearer understanding of the strengths and limitations inherent in each approach. While bulk sequencing offers a panoramic view of gene expression, it lacks the granularity to discern the contributions of individual cell types to HF. In contrast, SCS provides a high‐resolution lens through which to observe the intricate dynamics of the heart's cellular landscape. Reproducibility is a critical consideration in SCS research. Discussing common pitfalls and best practices in data generation and analysis is essential for maintaining the integrity of findings and ensuring that they can be replicated across different studies and laboratories.

In conclusion, single‐cell studies have provided invaluable insights into the mechanisms of HF and have identified new therapeutic targets. The integration of these findings into clinical practice holds great promise for revolutionizing the management of HF and enhancing patient outcomes. As we continue to refine our understanding of HF at the single‐cell level, we are poised to make significant strides in the prevention, diagnosis, and treatment of this debilitating condition.

## Conflict of Interest

The authors declare no conflict of interest.

## Supporting information

Supporting Information

Supplemental Table 1

Supplemental Table 2
