# Supplementary material for: From Cell to Gene: Deciphering the Mechanism of Heart Failure With Single‐Cell Sequencing
Source: Adv Sci (Weinh). 2024 Aug 19;11(39):2308900. doi: 10.1002/advs.202308900 (PMC11497092; doi:10.1002/advs.202308900)
Supplement: Supplementary file 1 — Supporting Information [file ADVS-11-2308900-s003.docx]

## Supplemental 1

## 1 Single-cell DNA amplification

There are several main methods for whole-genome amplification[1]. Methods based on thermal cycling PCR, including primer extension preamplification (PEP), degenerate oligonucleotide-primed PCR (DOP-PCR), and ligation-mediated PCR amplification (LM-PCR). The primer extension preamplification (PEP) emerged earlier and used a random primer containing 15 bases, annealed at 37°C, extended at 55°C, and so on, to amplify the entire genomic DNA randomly [2-4]. The DOP-PCR utilizes 6bp degenerate oligonucleotides at the 3' end and normal random primers at the 5' end. The 3' end of these primers can randomly bind to genomic DNA, enabling amplification of the entire genome[5-7]. The PEP-PCR has low requirements for template DNA, but its random primers anneal at different efficiencies. The PCR process may introduce errors and non-specific amplification primers, resulting in amplification bias. Compared with PEP-PCR, DOP-PCR has a higher DNA yield, but when the initial template amount is low, the deletion rate of the gene locus is high, and the amplification bias is significant. In addition, the poor uniformity of amplification caused by exponential amplification worsens the coverage[8]. The uncertainty between primers and templates and the interaction between primers may lead to low sensitivity and a high error rate of whole genome amplification, which leads to the limitations of using these two methods in single-cell sequencing. LM-PCR involves the fragmentation and ligation of the template DNA to the linker, followed by full amplification PCR[9]. While PicoPLEX and multiple annealing and looping-based amplification cycles (MALBAC) techniques combine PCR and isothermal replacement methods. Multiple displacement amplification (MDA) techniques use a set of random primers with the same temperature under the action of Phi29[10]. The linear amplification via transposon insertion (LIANTI) technique[11] uses transposons to chop DNA randomly so DNA samples can be linearly amplified. Before conventional PCR, MALBAC and LIANTI techniques effectively suppressed amplification preferences and improved genome-wide coverage at amplification by designing starting isothermal amplicons to form loops and transposons to be widely distributed in the genome.

MALBAC and MDA methods are the two predominant single-cell DNA amplification methods. ① MALBAC consists of two phases: linear MDA preamplification and PCR amplification. The MALBAC preamplification phase consists of multiple "anneal-extend-denaturation-cyclization" steps, in which single-cell genomic DNA is first unstranded at 94°C into single-stranded DNA molecules. Then, amplification begins with random primers containing a standard 27 nucleotide sequence and eight variable nucleotides, and n primers are uniformly hybridized to the template at 0°C. Variable-length half-amplicons were generated at 65°C using Phi29 polymerase with strand-substitution activity and followed by dissociation from the template at 94°C. Temperature cycling to 58°C allowed the entire amplicon to form a loop structure, thus preventing further amplification and cross-hybridization. In the successive five temperature cycles, after cyclization of the intact amplicon, the single-stranded amplicon and genomic DNA are used as templates to generate intact amplicons and additional half-amplicons, respectively. For the intact amplicon, the 3′ end is complementary to the 5′ end sequence, and the two ends hybridize to form circular DNA. After 5 cycles of linear preamplification, the intact amplicon is amplified by PCR index using a standard 27-nucleotide sequence as a primer. The MALBAC method is ingenious in that it anneals at 58°C, allowing intra-strand hybridization at both ends of the intact amplification product, which does not trigger amplification starting at the 3'. It avoids the self-exponential amplification of the complete amplification product and achieves linear amplification. Secondly, multiple amplicons can be amplified on a single template by utilizing the forechain removal function of Phi29 polymerase. In each of the latter five rounds of amplification, the original template was allowed to be amplified again with multiple amplicons, ensuring genome-wide coverage. After m cycles, the number of complete amplification products obtained is m × n^2^, proportional to the number of amplifications m, ensuring linear amplification with high efficiency [1, 10, 12]. The MALBAC method is simple to operate, has high amplification efficiency and sensitivity, good genome coverage, and amplification uniformity, and is friendly to rare and highly concordant samples, which has significant advantages for subsequent whole genome analysis. The disadvantage is that amplification bias will likely occur when the template copy number is very low, and non-specific amplification may occur [12]. ② MDA method, an amplification technique based on isothermal reaction. The Phi29 DNA polymerase with strong strand replacement activity and exonuclease activity replicates simultaneously at multiple sites of DNA, synthesizing DNA along the template strand, replacing the complementary strand of the template strand, which in turn serves as a template for further amplification, thus forming a set of super-amplified branching networks and obtaining multiple overlapping copies of the template [13]. The MDA method, a more straightforward and efficient method than MALBAC, does not require special instruments, the amount of amplified product is stable, and the fragments are long (>10kb), which is conducive to improving single cell whole genome coverage [14]. The disadvantages are the high template quality required, the possible generation of non-specific products, and the potential for library preference [10, 15, 16].

## 2 Single-cell RNA amplification

To achieve single-cell mRNA sequencing without significant PCR bias, increasing mRNA increments by several million-fold may be necessary, owing to the considerably lower mRNA content in cells. Instead of libraries containing substantial amounts of ribosomal RNA (rRNA) sequences, obtaining libraries consisting of mRNA is feasible. It should be noted that more than 95% of the total RNA in cells is constituted by rRNA, with mRNA accounting for only 2-3%. Consequently, if a non-selective reverse transcription, amplification, library construction, and sequencing approach is adopted, most resulting sequences will not offer pertinent biological information about mRNA. As a result, PCR or multiplexed linear amplification techniques are generally employed for their amplification. SMART-seq [17], SMRT-seq2 [18], CEL-seq [19], CEL-seq2 [20], and STRTseq [21], which are based on full-length RNA sequencing and high-throughput microfluidics-based methods, such as DROP-seq [22] and 10 × Genomics to achieve intracellular mRNA amplification. Following is a brief description of the process for representative applications, ①SMART methods; the core technology underlying the SMART approach involves the strategic design of two specialized primers. The initial primer in reverse transcription encompasses a generic sequence featuring a poly(T) structure at its center. This configuration enables precise binding with the poly(A) tail found in the mRNA. Consequently, during reverse transcription facilitated by MMLV reverse transcriptase, the resulting cDNA possesses a few additional C bases at its 3' end. Subsequently, the upstream primer, comprising three non-deoxygenated G bases at its 3' end, complements the C base present in the cDNA. Once again, under the auspices of MMLV reverse transcriptase, a novel double-stranded DNA molecule is synthesized, utilizing the cDNA as a template. This DNA molecule is endowed with PCR primers at both termini, enabling conventional PCR amplification to yield substantial DNA. ② 10 × Genomics technology combines a single-cell suspension, prepared gel beads with barcode, unique molecular identifiers (UMI), and Poly(dT)VN reverse transcription primers. The oil droplets are added to different chambers of 10×Chromium Chip B to form water-in-oil droplets by the "double cross" crossover system, followed by the release of mRNA from the cells. Under substrates, primers, and reverse transcriptase, mRNA can be reverse-transcribed into cDNA with UMI, and then amplification and library construction proceed. The UMI technology can effectively compensate for the preference for PCR amplification in the library construction process due to the distortion of transcript expression level with low initial quantity. This method applies well to the quantitative analysis of low-abundance genes in the transcriptome [23, 24].

**3.1 Quality Control and matrix generation.** Single-cell sequencing technology incorporating barcoding enables cost-effective and massively parallel processing. Before analysis, filtering based on UMIs is necessary to eliminate invalid barcodes representing incomplete individual and non-single-cell information. Generating a count matrix is essential for subsequent downstream analyses and biological insights. The STAR algorithm efficiently identifies splice junctions and aligns sequenced fragments to the reference genome in a non-contiguous manner. This widely adopted approach aligns the raw sequencing data to publicly available platform sequences, resulting in comprehensive gene expression profiles[25]. Another gained popularity in the single-cell field is Kallisto, which constructs k-compatibility class from the reads and performs pseudo alignment based on the de Bruijn graph[26]. Each barcode is filtered for the number, gene count, and percentage of mitochondrial genes. Generally, the mitochondrial gene content is less than 5%, but cardiac muscle cells are significantly higher than other cells. Mitochondrial transcripts accounted for approximately 30% of total mRNA in the human heart, and in adult mouse cardiomyocytes, they ranged from 58% to 86% [27, 28]. Mitochondrial percentage threshold settings and adjusting are determined according to tissue type and the actual situation.

**3.2 Standardization.** The number of valuable reads obtained from sequencing varied between cells and required correction. There are different types of normalization methods for single-cell data, of which the normalization of data by Scran and Seurat is the more popular method[29, 30]. Other normalization methods include SCtransform, SCnorm, and BayNorm normalization methods[31-33]. After the normalization, the logarithmic transformation is performed.

**3.3 Feature Selection.** Identify highly variable genes with the strongest biosignal relative to technical noise, thus limiting the downstream analysis to the most informative genes, reducing the effect of dimensionality, and simplifying analysis[34].

**3.4 Dimensionality reduction, data visualization, and clustering analysis.** The scRNA-seq data are multidimensional and require dimensionality reduction for visualization and analysis. PCA is a linear transformation that preserves the Euclidean distance between cells, and principal components can be mapped to biological covariates to understand their efficacy. However, linear transformations such as PCA cannot capture cell relationships due to high deletion and noise, so some nonlinear dimensionality reduction methods, such as t-SNE and UMAP, are usually used. t-SNE focuses on capturing local similarity but discards global structure. UMAP captures underlying data structures better, aggregates data across more than two dimensions, and runs fast. However, t-SNE and UMAP highly depend on user-selected parameters susceptible to the results. Further clustering analysis is required due to the dropping distance between cells using the above two methods. Clustering is an unsupervised machine-learning process based on distance matrices. Cell clustering allows cells to be grouped to infer cell types based on gene expression similarity, including the Louvain and Leiden detection algorithms. After dimensionality reduction, the data is ready for downstream analysis.

**3.5 Cell trajectory analysis.** Changes in cell components are strongly correlated with disease states and differ in the same organ, especially in highly heterogeneous complex organs such as the heart. Trajectory analysis captures the significant characteristics of cells in the transformation process and can identify cell identity and explore the process of branching and differentiation and changes in biological function.

**3.6 Gene differential expression, regulatory networks, and cell interactions.** In recent years, differential expression analysis methods for scRNA-seq have been developed and applied successively, such as MAST, SCDE, DEsingle, Census, and BCseq[35]. The Seurat can be used for differential expression analysis using different models. To correlate information from scRNA-seq datasets with phenotypic variables, regression-based models can correlate gene expression changes [30]. Gene level analysis can also be performed for gene set enrichment analysis used in bulk-RNAseq such as GO, KEGG, and Reactome or weighted correlation network analysis (WGCNA). In addition, ligand-receptor analysis is performed using paired gene tags, and interactions between cell clusters are inferred from the expression of the receptor and its homologous ligands.

Several challenges are encountered in acquiring single-cell suspension, particularly when dealing with large and fragile myocardial cells. Additionally, the process of digesting and dissociating the pathological remodeling site proves to be difficult. Achieving linear amplification is crucial during the amplification phase, ensuring whole-genome coverage and optimizing efficiency. Moreover, the library construction process faces challenges like mRNA degradation and inefficient reverse transcription. Although flow cytometry, integrated fluid pathways systems, and other plate-based platforms are commonly employed for larger and fragile cells like cardiomyocytes, they can only process a limited number of cells simultaneously. However, gradual advancements in technology and platform development are expected to overcome these issues[36]. The ICELL8 system and related newer approaches enable high-throughput processing of hundreds of single cells without typical microfluidics cell size or the imaging limitations of droplet-based systems [37, 38]. Adding a known concentration of synthetic mRNAs to the transcript synthesis is one option for calibrating systematic errors to achieve standardized analysis[39]. scRNA-seq is now widely used in cardiac-related studies, and carefully selecting the most appropriate platform is crucial to meet the success of subsequent research.

References

[1] X. Wang, Y. Liu, H. Liu, W. Pan, J. Ren, X. Zheng, Y. Tan, Z. Chen, Y. Deng, N. He, Recent advances and application of whole genome amplification in molecular diagnosis and medicine, MedComm, 3 (2022) e116.

[2] A. Maciejewska, J. Jakubowska, R. Pawlowski, Different whole-genome amplification methods as a preamplification tool in Y-chromosome Loci analysis, The American Journal of Forensic Medicine and Pathology, 35 (2014) 140-144.

[3] J. Traeger-Synodinos, Pre-implantation genetic diagnosis, Best Practice & Research Clinical Obstetrics & Gynaecology, 39 (2017) 74-88.

[4] R.S. Lasken, M. Egholm, Whole genome amplification: abundant supplies of DNA from precious samples or clinical specimens, Trends in biotechnology, 21 (2003) 531-535.

[5] C. Gawad, W. Koh, S.R. Quake, Single-cell genome sequencing: current state of the science, Nature Reviews Genetics, 17 (2016) 175-188.

[6] P. Mendez, L.T. Fang, D.M. Jablons, I.-J. Kim, Systematic comparison of two whole-genome amplification methods for targeted next-generation sequencing using frozen and FFPE normal and cancer tissues, Scientific reports, 7 (2017) 4055.

[7] L. Huang, F. Ma, A. Chapman, S. Lu, X.S. Xie, Single-cell whole-genome amplification and sequencing: methodology and applications, Annual review of genomics and human genetics, 16 (2015) 79-102.

[8] P.-Y. Kwok, Making ‘random amplification’predictable in whole genome analysis, TRENDS in Biotechnology, 20 (2002) 411-412.

[9] A. Masny, A. Płucienniczak, Ligation mediated PCR performed at low denaturation temperatures--PCR melting profiles, Nucleic Acids Res, 31 (2003) e114.

[10] L. Huang, F. Ma, A. Chapman, S. Lu, X.S. Xie, Single-Cell Whole-Genome Amplification and Sequencing: Methodology and Applications, Annu Rev Genomics Hum Genet, 16 (2015) 79-102.

[11] C. Chen, D. Xing, L. Tan, H. Li, G. Zhou, L. Huang, X.S.J.S. Xie, Single-cell whole-genome analyses by Linear Amplification via Transposon Insertion (LIANTI), 356 (2017) 189-194.

[12] C. Zong, Multiple Annealing and Looping-Based Amplification Cycles (MALBAC) for the Analysis of DNA Copy Number Variation, Genomic Mosaicism in Neurons and Other Cell Types2017, pp. 133-142.

[13] N. Liu, L. Liu, X. Pan, Single-cell analysis of the transcriptome and its application in the characterization of stem cells and early embryos, Cellular and molecular life sciences, 71 (2014) 2707-2715.

[14] J.R. Nelson, Random‐primed, Phi29 DNA polymerase‐based whole genome amplification, Current Protocols in Molecular Biology, 105 (2014) 15.13. 11-15.13. 16.

[15] A.M. Sidore, F. Lan, S.W. Lim, A.R. Abate, Enhanced sequencing coverage with digital droplet multiple displacement amplification, Nucleic acids research, 44 (2016) e66-e66.

[16] R.S.J.B.S.T. Lasken, Genomic DNA amplification by the multiple displacement amplification (MDA) method, 37 (2009) 450-453.

[17] D. Ramsköld, S. Luo, Y.-C. Wang, R. Li, Q. Deng, O.R. Faridani, G.A. Daniels, I. Khrebtukova, J.F. Loring, L.C.J.N.b. Laurent, Full-length mRNA-Seq from single-cell levels of RNA and individual circulating tumor cells, 30 (2012) 777-782.

[18] S. Picelli, O.R. Faridani, Å.K. Björklund, G. Winberg, S. Sagasser, R. Sandberg, Full-length RNA-seq from single cells using Smart-seq2, Nature protocols, 9 (2014) 171-181.

[19] T. Hashimshony, F. Wagner, N. Sher, I.J.C.r. Yanai, CEL-Seq: single-cell RNA-Seq by multiplexed linear amplification, 2 (2012) 666-673.

[20] T. Hashimshony, N. Senderovich, G. Avital, A. Klochendler, Y. De Leeuw, L. Anavy, D. Gennert, S. Li, K.J. Livak, O.J.G.b. Rozenblatt-Rosen, CEL-Seq2: sensitive highly-multiplexed single-cell RNA-Seq, 17 (2016) 1-7.

[21] S. Islam, U. Kjällquist, A. Moliner, P. Zajac, J.-B. Fan, P. Lönnerberg, S.J.G.r. Linnarsson, Characterization of the single-cell transcriptional landscape by highly multiplex RNA-seq, 21 (2011) 1160-1167.

[22] J. Bageritz, G. Raddi, Single-cell RNA sequencing with drop-seq, Single Cell Methods: Sequencing and Proteomics, (2019) 73-85.

[23] S.A. MacParland, J.C. Liu, X.-Z. Ma, B.T. Innes, A.M. Bartczak, B.K. Gage, J. Manuel, N. Khuu, J. Echeverri, I.J.N.c. Linares, Single cell RNA sequencing of human liver reveals distinct intrahepatic macrophage populations, 9 (2018) 1-21.

[24] D.A. Jaitin, E. Kenigsberg, H. Keren-Shaul, N. Elefant, F. Paul, I. Zaretsky, A. Mildner, N. Cohen, S. Jung, A. Tanay, Massively parallel single-cell RNA-seq for marker-free decomposition of tissues into cell types, Science, 343 (2014) 776-779.

[25] A. Dobin, C.A. Davis, F. Schlesinger, J. Drenkow, C. Zaleski, S. Jha, P. Batut, M. Chaisson, T.R. Gingeras, STAR: ultrafast universal RNA-seq aligner, Bioinformatics, 29 (2013) 15-21.

[26] N.L. Bray, H. Pimentel, P. Melsted, L. Pachter, Near-optimal probabilistic RNA-seq quantification, Nature biotechnology, 34 (2016) 525-527.

[27] T. Ilicic, J.K. Kim, A.A. Kolodziejczyk, F.O. Bagger, D.J. McCarthy, J.C. Marioni, S.A.J.G.b. Teichmann, Classification of low quality cells from single-cell RNA-seq data, 17 (2016) 1-15.

[28] M.M. Gladka, B. Molenaar, H. De Ruiter, S. Van Der Elst, H. Tsui, D. Versteeg, G.P. Lacraz, M.M. Huibers, A. Van Oudenaarden, E. Van Rooij, Single-cell sequencing of the healthy and diseased heart reveals cytoskeleton-associated protein 4 as a new modulator of fibroblasts activation, Circulation, 138 (2018) 166-180.

[29] A. Lun, Using scran to analyze single-cell RNA-seq data, dim, 20064 (2019) 1728.

[30] T. Stuart, A. Butler, P. Hoffman, C. Hafemeister, E. Papalexi, W.M. Mauck, Y. Hao, M. Stoeckius, P. Smibert, R. Satija, Comprehensive integration of single-cell data, Cell, 177 (2019) 1888-1902. e1821.

[31] A.S. Booeshaghi, I.B. Hallgrímsdóttir, Á. Gálvez-Merchán, L. Pachter, Depth normalization for single-cell genomics count data, bioRxiv, (2022) 2022.2005. 2006.490859.

[32] R. Bacher, L.-F. Chu, N. Leng, A.P. Gasch, J.A. Thomson, R.M. Stewart, M. Newton, C. Kendziorski, SCnorm: robust normalization of single-cell RNA-seq data, Nature methods, 14 (2017) 584-586.

[33] W. Tang, F. Bertaux, P. Thomas, C. Stefanelli, M. Saint, S. Marguerat, V. Shahrezaei, bayNorm: Bayesian gene expression recovery, imputation and normalization for single-cell RNA-sequencing data, Bioinformatics, 36 (2020) 1174-1181.

[34] T.S. Andrews, V.Y. Kiselev, D. McCarthy, M.J.N.p. Hemberg, Tutorial: guidelines for the computational analysis of single-cell RNA sequencing data, 16 (2021) 1-9.

[35] T. Wang, B. Li, C.E. Nelson, S. Nabavi, Comparative analysis of differential gene expression analysis tools for single-cell RNA sequencing data, BMC Bioinformatics, 20 (2019) 40.

[36] D.T. Paik, S. Cho, L. Tian, H.Y. Chang, J.C. Wu, Single-cell RNA sequencing in cardiovascular development, disease and medicine, Nature Reviews Cardiology, 17 (2020) 457-473.

[37] L.D. Goldstein, Y.-J.J. Chen, J. Dunne, A. Mir, H. Hubschle, J. Guillory, W. Yuan, J. Zhang, J. Stinson, B. Jaiswal, Massively parallel nanowell-based single-cell gene expression profiling, BMC genomics, 18 (2017) 1-10.

[38] O. Shomroni, M. Sitte, J. Schmidt, S. Parbin, F. Ludewig, G. Yigit, L.C. Zelarayan, K. Streckfuss-Bömeke, B. Wollnik, G. Salinas, A novel single-cell RNA-sequencing approach and its applicability connecting genotype to phenotype in ageing disease, Scientific Reports, 12 (2022) 4091.

[39] A.T. Lun, F.J. Calero-Nieto, L. Haim-Vilmovsky, B. Göttgens, J.C. Marioni, Assessing the reliability of spike-in normalization for analyses of single-cell RNA sequencing data, Genome research, 27 (2017) 1795-1806.
